# A new species of the genus ***Duvalius*** sg. ***Neoduvalius*** from Montenegro with taxonomical remarks on the genus ***Duvalius*** (Coleoptera, Carabidae, Trechini)

**DOI:** 10.3897/zookeys.278.4650

**Published:** 2013-03-21

**Authors:** Roman Lohaj, Dávid Čeplík, Ján Lakota

**Affiliations:** 1 nstitute of Forensic Sciences, Sklabinská 1, SK-812 72 Bratislava,, Slovakia; 2 Južná trieda 13, 040 01 Košice, Slovakia; 3 Generála Miloša Vesela 5304/1, SK-034 01 Ružomberok, Slovakia

**Keywords:** *Duvalius (Neoduvalius) gejzadunayi* sp. n., *Serboduvalius*, *Rascioduvalius*, *Javorella*, *Curcicia*, subterranean environment, Coleoptera, Carabidae, Trechinae, Duboki potok cave, Rožaje, Montenegro, Serbia, taxonomy, new synonymy

## Abstract

*Duvalius* (sg. *Neoduvalius*) *gejzadunayi* **sp. n.** from Pećina u Dubokom potoku cave ( Donje Biševo village near Rožaje, Montenegro), the first known representative of this subgenus from the territory of Montenegro is described, illustrated and compared with the related species of the subgenus *Neoduvalius* Müller, 1913. This new species is characterised by depigmented, medium sized body, totally reduced eyes, deep and complete frontal furrows, 3–4 pairs of discal setae in third elytral stria, as well as by the shape of aedeagus. Data on the distribution and the ecology of this remarkable species, as well as a check-list of the subgenus *Neoduvalius* are also provided. Recently described genera *Serboduvalius* Ćurčić, S. B. Pavićević & Ćurčić, B.P.M., 2001, *Rascioduvalius* Ćurčić, S. B. Brajković, Mitić & Ćurčić, B.P.M., 2003, *Javorella* Ćurčić, S. B. Brajković, Ćurčić, B.P.M. & Mitić, 2003 and *Curcicia* Ćurčić, S. B. & Brajković, 2003 are regarded as junior synonyms of the genus *Duvalius* Delarouzée.

## Introduction

The Dinaric mountain chain, and the Balkan peninsula as a whole, are an impressive hotspot of subterranean biodiversity (see, as examples, Pretner 1969, 1973, 1977, Bedek et al. 2006, Gottstein et al. 2002, Jalžić 1984, 1994, Jalžić et al. 2010). Despite the fact that the cave fauna of this region has systematically been studied for more than one and half centuries new taxa are frequently described. Intensive biospeleological research performed in the Dinaric karst region during last twenty years has led to the discovery of many new subterranean trechine taxa, species and also genera (Monguzzi 1993, Casale and Guéorguiev 1994, Casale and Jalžić 1999, Quéinnec 2008, Quéinnec and Pavićević 2008, Quéinnec et al. 2008, Lohaj and Jalžić 2009, Lohaj and Lakota 2010, Lakota et al. 2010, Casale et al. 2012, Lohaj and Mlejnek 2012).

During biospeleological research in the caves of the vicinity of Rožaje (Montenegro) undertaken by members of association Biospeleologica Slovaca (D. Čeplík, G. Dunay, J. Lakota and R. Lohaj) in 2009, a new species of the trechine genus *Duvalius* belonging to the sg. *Neoduvalius* was discovered. This new species is described below.

## Material and methods

The morphological features of beetles were examined using Olympus SZ 60 and MBS 10 stereo-microscopes. Male and female genitalia were dissected, cleaned and mounted in Euparal® on transparent slides under the examined specimens. Macrophotographs were taken using a Canon 5D mark II camera. Photographs of genitalia were taken using microscope Leitz Ergolux with Nikon Colpix E4500 digital camera attached and were completed using Helicon Focus software program.

### Measurements

TLsourcetotal body length (measured from the anterior margin of clypeus to the apex of elytra)


Lsourceoverall length, from apex of mandibles to apex of elytra, measured along the suture


HLsourcehead length (measured from the anterior margin of the clypeus to the neck constriction)


HWsourcemaximum width of head


HL/HWsourceratio length of head/ maximum width of head


PLsourcepronotum length (measured along the median line)


PWsourcemaximum width of pronotum, as greatest transverse distance


PL/PWsourceratio length of pronotum/maximum width of pronotum


ELsourceelytral length (as linear distance measured along the suture from the elytral base to the apex)


EWsourcemaximum width of elytra


EL/EWsourceratio length of elytra/maximum width of elytra


## Collections

HPMcollection of Hrvatski Prirodoslovni Muzej, Zagreb, Croatia (B. Jalžić)


NMPcollection of Národní Muzeum Praha, Czech republic


ZSMcollection of Zoologische Staatssammlung Munich, Germany


CACprivate collection of Achille Casale, Torino, Italy


CDCprivate collection of Dávid Čeplík, Košice, Slovakia


CGDprivate collection of Gejza Dunay, Kráľovce, Slovakia


CAGprivate collection of Artur Gitzen, Neuhofen, Germany


CJLprivate collection of Ján Lakota, Ružomberok, Slovakia


CRLprivate collection of Roman Lohaj, Pezinok, Slovakia


CVZprivate collection of Vladimír Zieris, Pardubice, Czech republic


hwhandwritten


pprinted


### Forward slash indicates separate labels

The following species have been also studied:

*Duvalius (Neoduvalius) eurydice* (Schaufuss, 1881): 1♂ labelled: „Croatia (hw)/Coll. Geittner (p)“ (ZSM); 1♀ „05028 (p)/Raduć. (p) 25.VII. (hw)“ (ZSM); 1♂ „Croatia (p)/*eurydice* (hw)/Sammlung Dr.K.Daniel (p)“ (ZSM); 1♂ „Croatia (p)/Collection Strasser (p)“ (ZSM).

*Duvalius (Neoduvalius) guidononveilleri* Janák & Moravec, P., 2008: 1♂ Paratype labelled: „Jugoslavija, Srbija, Divčibare–sedlo, 750-900 m, 20.5.1991, V.Zieris lgt.“ (CVZ).

*Duvalius (Neoduvalius) klimeschi* (Winkler, 1914): 1♀ labelled: „Dinar Alpen Troglav–Geb. A.Winkler (p)/*Neoduv. troglavensis* Wkl. (hw)“ (ZSM).

*Duvalius (Neoduvalius) kodrici* (Scheibel, 1938): 1♂1♀ labelled: „Pljesevica grupe NO Lika Cro, L.Weirather, Insbruck (p)/Höhle Nr. (p) 658 (hw) grundbuch, Weirather (p)/*Neoduvalius opermanni* Schbl. (hw)“ (ZSM, CAC)

*Duvalius (Neoduvalius) langhofferi* (Csiki, 1913): 1♀ labelled: „Ogulin, Tamnica potok, Donje Dubrave, 22.06.2008, B. Jalžić (hw)“ (HPM); 1♀ „Tamnica potok, D.Dubrave, Kordun, 2.03.1985, leg. Jalžić (hw)“ (HPM); 1♀ „Kordun, Mateško selo, Mijatova jama, 15. trav. 1979, Rada“ (HPM); 1♀ „Kordun, Mateško selo, Mijatova jama“ (HPM).

*Duvalius (Neoduvalius) opermanni* Scheibel, 1933: 2♂♂ labelled: „CROATIA, Ogulin, Slunj, Stara Kršlja, Dumenčića špilja cave, 21.5.2012 resp. 19.9.2012, J. Lakota, B. Jalžić lgt.“ (CJL, HMP).

*Duvalius (Neoduvalius) reitteri* (Miller, 1881): 1♂ labelled: „Croatia Stava. (hw)/*Reitteri* Mil.. (hw)/Coll. Mihók (p)/*Neoduvalius reitteri* Mill. (hw) Det.Dr.Bokor E. (p)“ (ZSM); 1♀ „Senjsko Bilo Cro., Winkler (p)“ (ZSM); 1♂ „Senjsko Bilo Cro., Winkler (p)/*Anophthalmus reitteri* Mill. (hw)“ (ZSM); 1♂ „*A.Reitteri* Mit. Croatien (hw)“ (ZSM); 1♂ „Ostrovica Geb. 8.7.89 (hw)/*Reitteri* (hw)“ (ZSM); 1♂ „Mogorice (hw)/Croatia (hw)/Reitteri Mill. (hw)/ Sammlung Dr.K.Daniel (p)“ (ZSM); 1♀ „Croatien Likaer Grotten Reitter 79 (p)/Sammlung Müller (p) (ZSM); 8♂♂, 7♀♀: „Bosnia, 1.8.2009, Bosanski Petrovac, Dragišića pećina cave, individually, D.Čeplík, R. Lohaj lgt.“ (CDC, CRL); 6♂♂, 7♀♀: „Croatia, Lika, Gospić, Mogorić willage env., Pčelina špilja cave, 22.5.2012, individually, D.Čeplík, B. Jalžić, J. Lakota, R. Lohaj lgt.“ (HPM, CDC, CJL, CRL).

*Duvalius (Neoduvalius) schatzmayri* (J. Müller, 1912): 1♀ labelled: „Mračna pećina Prolog Bos. Novak 17.8.89 (p)/*Neoduvalius schatzmayri* J. Müll. (hw) det. F. Stöcklein 1953 (p)“ (ZSM).

*Duvalius (Neoduvalius) starivlahi* Guéorguiev, B.V., Čurčić, S.B. & Čurčić, B.P.M., 2000: 1♀ labelled: Serbia, Hadži Prodanova Pećina cave, Raščići, Ivanjica, Leg. B. Mitić, 29.05.2003“ (CRL), 1♂ the same data, but Leg. S. Čurčić, 28.05.2008 (CAG).

*Duvalius (Neoduvalius) styx* (Apfelbeck, 1904): 1♂ labelled: „Mrzla pec. Plitvic. jez. (hw)/Weir. VII.39 H.658 (hw)“ (ZSM).

## Result

### Duvalius (Neoduvalius) gejzadunayi sp. n.

urn:lsid:zoobank.org:act:707EAD03-4EE3-4A13-9592-1178ADA5938C

http://species-id.net/wiki/Duvalius_gejzadunayi

Figs 1, 3 5, 6, 7

#### Material examined.

Holotype male labelled as follows: „MONTENEGRO, Rožaje, Donje Biševo, Pećina u Dubokom potoku cave, 17.4.–11.8. 2009 traps, R.Lohaj, D.Čeplík & G.Dunay lgt.“ (white label, printed) / „HOLOTYPUS *Duvalius (Neoduvalius) gejzadunayi* sp. n. R. Lohaj, D. Čeplík & J.Lakota det. 2011“ (red label, printed), (NMP). Paratypes: 6 ♂♂ 7 ♀♀, the same data as Holotype (CDC, CGD, CAG, CJL, CRL, CVZ). All paratypes are labelled with white, printed locality labels and with red printed labels „PARATYPUS *Duvalius (Neoduvalius) gejzadunayi* sp.nov. R. Lohaj, D.Čeplík & J.Lakota det. 2011“.

#### Diagnosis.

**Figures 1–4.**
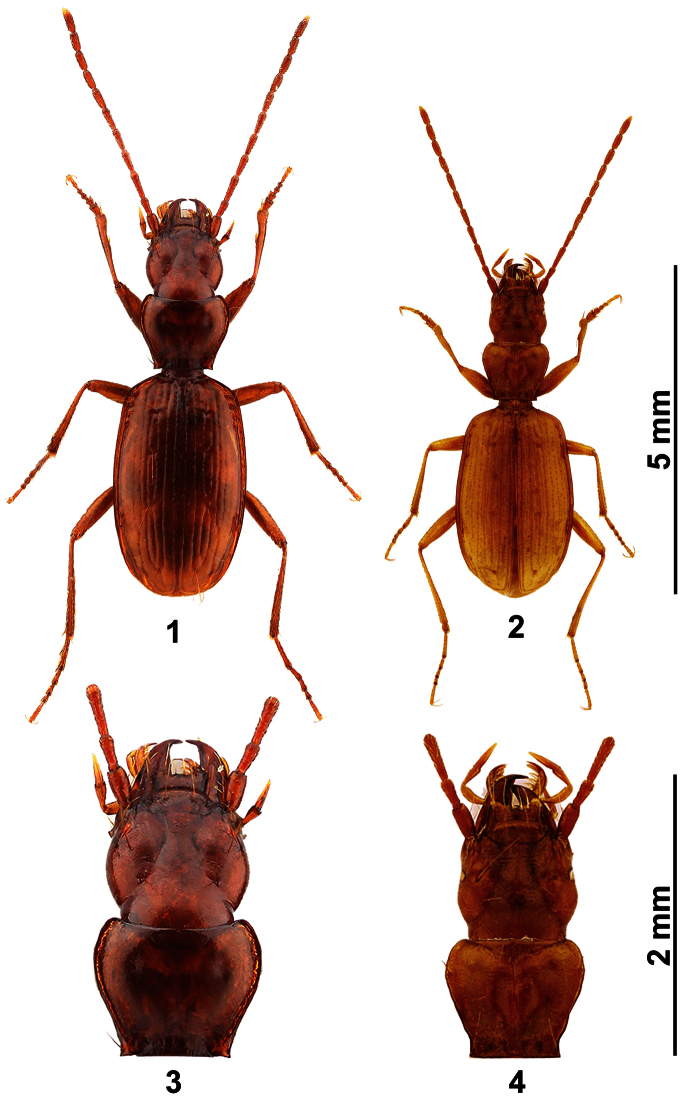
**1, 3** *Duvalius (Neoduvalius) gejzadunayi* sp. n., Holotype male, Duboki potok cave **2, 4** *Duvalius (Neoduvalius) starivlahi* Guéorguiev et al. 2000, Topotype male, Hadži Prodanova pećina cave **1, 2** habitus, dorsal view **3, 4** detail of head and pronotum.

A medium sized (L 5.5–6.3 mm), glabrous, depigmented, anophthalmous trechine species with the character states of the genus *Duvalius* Delarouzée, 1859 of subgenus *Neoduvalius* J. Müller, 1913 (Fig. 1). Colour reddish-brown, pronotum cordiform, transverse, head large and rounded, with deep, complete frontal furrows (Fig. 3), elytral stria 3 with 3–4 discal setigerous punctures. Close to *Duvalius (Neoduvalius) starivlahi* Guéorguiev, Ćurčić, S.B. & Ćurčić, B.P.M, 2000, from which is distinguished by several different morphological features (see: Discussion).

#### Description.

TL 5.2–6.0 mm (HT 5.3 mm). Colour reddish-brown, legs, antennae and palpi paler. Glabrous, shiny, head and pronotum with distinct isodiametric microsculpture, microsculpture of elytra with isodiametric and transverse meshes.

Head large, rounded, neck markedly distinct, HL 0.85- 0.97 mm (HT 0.87 mm), HW 1.05–1.22 mm (HT 1.08 mm), slightly narrower than pronotum, index HL/HW 0.79 - 0.81 (HT 0,80), glabrous, with distinct isodiametric microsculpture. Frontal furrows deep, complete, reaching neck constriction. Head with two pairs of long supraorbital setae, anterior pair behind middle of head length, posterior pair at hind part of head near the neck. Mandibles relatively long and slender, acutely pointed, the right one with tridentate basal teeth. Clypeus with 2 pairs of setae, labrum with three pairs. Eyes completely reduced, in some specimens present as a dark spot. Antennae long and slender, reaching almost half of elytral length, covered with dense decumbent pubescence, antennomere 3 longest, scape and antennomeres 5–10 nearly equally long.

Pronotum glabrous, slightly transverse, with maximum width in anterior fifth, PL 0.95–1.08 mm (HT 1.00 mm), PW 1.13–1.40 mm (HT 1.25 mm), index PL/PW 0.75–0.81 (HT 0.80), on base distinctly narrower than on anterior margin. Sides rounded, before hind angles sinuate, anterior angles rounded, obtuse, posterior sharply pointed. Lateral furrows well developed, deep, with two pairs of setae; anterolateral setae situated in the anterior fifth, basolateral pair before hind angles. Median furrow weakly marked, visible in middle of pronotum.

Elytra glabrous, elongate, almost parallel-sided, with maximum width in middle, EL 2.80–3.25 mm (HT 3.00 mm), EW 1.75–2.10 mm (HT 1.85 mm), index EL/EW 1.58–1.70 (HT 1.62), apically rounded. Shoulders well defined, forming obtuse angle, scutellum small, flat; single pair of basal scutellar setigerous pores present. Elytral striae 1–4 well developed, deep, striae 5 –7 vanished, reduced to rows of foveae. Elytral stria 3 with 3–4 discal setigerous punctures (formula 3 + 3, 3 + 4, 4 + 3 or 4 + 4) and pair of apical ones. Umbilicate series consists of 9 setae on both elytra, formula 4 humeral + 2 middle + 3 apical, humeral group of umbilicate pores aggregated. Ventrites 4–6 glabrous, each with pair of setae on their posterior margins, anal ventrite with pair of setae in males and females.

Legs long, slender, densely pubescent, protibiae with a deep longitudinal furrow on their dorsal side. First two tarsomeres of male protarsi distinctly dilated and toothed at their internal margins. Tarsal claws long and slender, pointed at apex.

**Figures 5–7.**
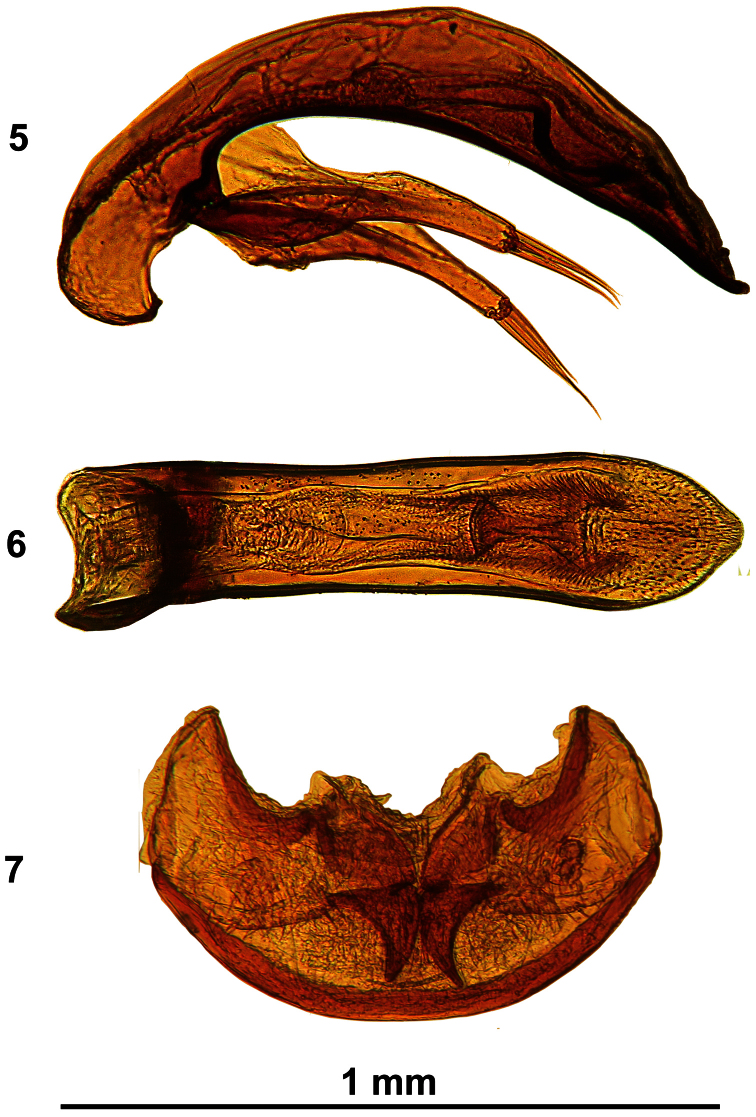
*Duvalius (Neoduvalius) gejzadunayi* sp. n., **5** aedeagus, left lateral aspect **6** aedeagus, dorsal aspect **7** female genitalia, ventral aspect

Aedeagus (Figs 5, 6) 0.91–1.00 mm long (HT 0.97 mm), median lobe in lateral aspect regularly curved and moderately narrowed apically. Endophallus with copulatory piece widely bilobed both at base and apex, with a reduced bundle of scales in middle. Apex obtuse, widely rounded dorsaly. Parameres slender, length of parameres about half of length of aedeagus, each paramere with four thick apical setae.

Female genitalia: (Fig. 7): without peculiar features, apical segments of gonostyli elongated and slender, regularly curved, at apex pointed, with two dorsal and two ventral spines.

#### Etymology.

Patronymic, dedicated to our dear friend Gejza Dunay (Kráľovce, Slovakia), member of our biospeleological expeditions on Balkans and one of the discoverers of this new species.

#### Distribution.

So far known only from the type locality, Duboki potok cave near Rožaje, Montenegro.

#### Topographic location and ecology.

Rožaje town is situated in the easternmost part of Montenegro and is surrounded by high rugged mountains exceeding 2000 m, notably Bjelasica Mts to the west, Hajla Mts and Suva planina Mts to the south and Mokra Gora Mts to the east. To the north is the more open Pešter Polje hill country.

Pećina u Dubokom Potoku cave is situated at 1180 m near the village of Dinje Biševo, 8km north of Rožaje, on the left side of a deep clough valley with the Duboki Potok (English = „deep creek“) draining it. The Duboki Potok is one of the left-side confluents of Ibar river, which flows from this area to the Black Sea. The cave is inactive (without water course) with entrance of ca 1 × 1,5 m, followed by spacious hall with wet, black,  clay-like humus floor, but deeper in the cave this changes to an ochre-brown sticky mud. The cave floor is littered throughout with stones, stalagmites and stalactites occur only in the furthest recesses of the cave. Total length of the cave is nearly 60 m. Type series of *Duvalius gejzadunayi* sp. n. was collected by traps baited with cheese and meat, placed in various parts of the cave.

##### Associated fauna

Araneae (det. A. Mock, Košice, Slovakia)

Dysderidae sp.

Pseudoscorpionidea (det. R. Ozimec, Zagreb, Croatia)

Neobisiidae:

*Neobisium (Blothrus) umbratile* Beier, 1938

Diplopoda (det. A. Mock, Košice, Slovakia)

Chordeumatidae:

*Melogona broelemenni* (Verhoeff)

Collembola (det. Ľ. Kováč, Košice, Slovakia)

Entomobryidae, Entomobryinae:

*Heteromurus nitidus* (Templeton, 1835)

*Verhoeffiella media* (Loksa et Bogojević, 1967)

Entomobryidae, Tomocerinae:

*Tomocerus* sp.

Arrhopalitidae:

*Arrhopalites principalis* Stach, 1945

Neelidae:

*Megalothorax* sp.

Coleoptera

Leiodidae:

*Rozajella jovanvladimiri* Ćurčić SB et al., 2007 (type locality)

Staphylinidae, Pselaphinae:

*Bryaxis* sp.

## Discussion

Subgenus *Neoduvalius* includes 20 described species (see Check-list bellow), small to medium sized (4–9 mm), depigmented, glabrous, with large head, prominent temporae, strongly reduced or totally absent eyes and third elytral interval with 2–5 setigerous pores, distributed in subterranean habitats from Croatia (Mala Kapela, Lika and Plješevica) through Bosnia & Herzegovina and Serbia to Montenegro (Rožaje).

*Duvalius gejzadunayi* sp. n. is closely related to *Duvalius starivlahi* Guéorguiev et al. 2000, described from Hadži-Prodanova pećina cave near Ivanjica, southwest Serbia. Both species are unique within subgenus *Neoduvalius*, characterised by peculiar combination of characters: (1) head with deep and complete frontal furrows, (2) elytral disc with 3–4 (exceptionally 5) setigerous pores. Complete frontal furrows are, except these two mentioned species, present only in *Duvalius (Neoduvalius) cvijici* Jeannel, which possess 2 elytral setigerous pores. Species with 3 elytral discal setae are shared also with *Duvalius (Neoduvalius) schatzmayri* Müller and *Duvalius (Neoduvalius) petraeus* Knirsch, but both species possess short, incomplete frontal furrows.

*Duvalius (Neoduvalius) starivlahi* and *Duvalius (Neoduvalius) gejzadunayi* sp. n. can be easily separated using the following key:

**Table:** 

1(2)	Head elongate, subglobose, frontal furrows very deep. Eyes reduced, composed of 8–20 ommatidia (Fig. 4). Aedeagus strongly arcuate, forming an angle of 90^o^, basal bulb large, apex from dorsal view sinuated and bent (see Guéorguiev et al. 2000). TL 5.1–5.7 mm. Serbia, Ivanjica	*Duvalius (Neoduvalius) starivlahi* Guéorguiev et al. 2000
2(1)	Head rounded, frontal furrows shallower, eyes totally absent (Fig. 3). Aedeagus strongly elongate, apex from dorsal view widely rounded (Figs 5, 6). TL 5.2–6 mm. Montenegro, Rožaje	*Duvalius (Neoduvalius) gejzadunayi* sp. n.

### Taxonomical notes on the genus *Duvalius*

During the period 2001–2003, four new Serbian genera: *Serboduvalius* Ćurčić SB et al., 2001 (based on *Serboduvalius dragacevensis* Ćurčić SB et al., 2001 and *Duvalius Neoduvalius*) *starivlahi* Guéorguiev et al. 2000)); *Rascioduvalius* Ćurčić SB et al., 2003 (based on *Duvalius (Neoduvalius) cvijici* Jeannel, 1923); *Javorella* Ćurčić SB et al., 2003 (based on *Duvalius Duvalius* s.str.) *suvoborensis* Pavićević and Popović, 2001) and *Curcicia* Ćurčić SB and Brajković, 2003, (based on *Duvalius Duvalius* s.str.) *bolei* Pretner, 1963) were erected based on species formerly included in the genus *Duvalius* s.l. (Ćurčić et al. 2001, Ćurčić et al. 2003a, 2003b, Ćurčić and Brajković 2003).

The aforementioned genera were based on morphological features that are present among many other species of various subgenera of *Duvalius* s.l., namely: flattened and reduced eyes (*Serboduvalius*, *Rascioduvalius*, *Javorella*);anophthalmy (*Curcicia*); presence of darkened eye border (*Serboduvalius*, *Rascioduvalius*, *Javorella*),presence of deep and complete frontal furrows (*Serboduvalius*, *Rascioduvalius*, *Javorella*), or incomplete frontal furrows (*Curcicia)*, convex or flat genae which are glabrous (*Serboduvalius*, *Javorella*, *Curcicia*);or finely pubescent (*Rascioduvalius*); first male tarsomere almost twice as long as wide or longer than wide (all genera); presence of 2 pairs of discal elytral setae (*Rascioduvalius*, *Javorella*, *Curcicia*); or 3 (rarely 2–4) pairs of discal elytral setae (*Serboduvalius*); specific position of humeral setae (all genera, note: humeral group of umbilical series in all these taxa is formed by 4 aggregated setae, as in all other *Duvalius* representatives); presence of longitudinal furrows on protibiae (*Serboduvalius*, *Rascioduvalius*, *Javorella*); absence of such furrow (*Curcicia*); specific shape of copulatory piece of median lobe of aedeagus (all genera).

Based on a thorough literature research in combination of specimen examination we conclude that the type species designated for genera *Serboduvalius*, *Rascioduvalius*, *Javorella* and *Curcicia* do not exhibit any autapomorphies or synapomorphies, respectively, which satisfactorily separate them from other representatives of *Duvalius* s.l. and warrant erection of new taxa. Therefore the following new synonyms are proposed:

*Duvalius* Delarouzée, 1859

= *Serboduvalius* Ćurčić, S. B., Pavićević & Ćurčić, B.P.M., 2001, **syn. nov.**

= *Rascioduvalius* Ćurčić, S. B., Brajković, Mitić & Ćurčić, B.P.M., 2003, **syn. nov.**

= *Javorella* Ćurčić, S. B. Brajković, Ćurčić, B.P.M., & Mitić, 2003, **syn. nov.**

= *Curcicia* Ćurčić S. B. & Brajković, 2003, **syn. nov.**

### Check-list of the genus *Duvalius* sg. *Neoduvalius*.

***Duvalius*** Delarouzée, 1859: 65 type species *Duvalius raymondi* Delarouzée, 1859, subgenus ***Neoduvalius*** Müller, J., 1913: 180 type species: *Anophthalmus Reitteri* L. Miller, 1881

Locality data given here is recorded in the language of the original text.

**1 *Duvalius (Neoduvalius) bradycephalus*** Jeannel, 1928: 549 (*Duvalius*, TL: Herzégovine)

 = *Duvalius (Neoduvalius) hercegovinensis* Knirsch, 1926c: 62 (*Neoduvalius*,TL: »Herzegovina«)

 Distribution: Bosnia & Herzegovina

**2 *Duvalius (Neoduvalius) cvijici cvijici*** Jeannel, 1923: 10 (*Duvalites*, TL: Murtenica planina, vers 1100m. d’alt., en forêt, comm. de Bela Rjeka, département d’Užice (Serbie occidentale))

 Distribution: Serbia (Užice, Murtenica plateau)

***Duvalius (Neoduvalius) cvijici stopicensis*** Jeannel, 1923: 11 (*Duvalites*, TL: grotte Stopića pećina, à Rožanstvo, département d’Užice (Serbie occidentale))

 Distribution: Serbia (Užice, Zlatibor Mts)

**3 *Duvalius (Neoduvalius) cuniculinus*** Knirsch, 1929: 86 (*Neoduvalius*, TL: Crna-gora (Herc.)

 Distribution: Bosnia & Herzegovina (Glavatićevo)

**4 *Duvalius (Neoduvalius) dragacevensis***Ćurčić , S.B., Pavičevć & Ćurčić, B.P.M., 2001: 53 (*Serboduvalius*, TL: Mala Pećina Cave, village Rti, near Kotraža, the Dragačevo Mts, southwestern Serbia)

 Distribution: Serbia (Ivanjica, Dragaćevo Mts)

 Note: This species was described based on set of 9 specimens (7 males, 2 females) collected in Mala pećina cave near willage Kotraža. This locality is situated ca 10 km from Hadži-Prodanova pećina cave, type locality for *Duvalius (Neoduvalius) starivlahi* Guéorguiev et al. Based on presented morphological differencies it is very probable that these two species are conspecific.

**5 *Duvalius (Neoduvalius) eurydice*** Schaufuss, 1881: 86 (*Anophthalmus*, TL: in cavernis Croatiae [=Špilja u Lici (=Špilje Like) (Bedek et al. 2006: 73)])

 Distribution: Croatia (Lika)

**6 *Duvalius (Neoduvalius) gejzadunayi* sp. n.,** TL: Montenegro, Rožaje, Donje Biševo, Pećina u Dubokom potoku cave

 Distribution: Montenegro (Rožaje)

**7 *Duvalius (Neoduvalius) guidononveilleri*** Janák & Moravec, P., 2008: 12 (*Duvalius*, TL: Jugoslavija, Srbija: Maljen, Divčibare-sedlo, 700-800m)

 Distribution: Serbia (Maljen plateau)

**8 *Duvalius (Neoduvalius) humerosus*** Knirsch, 1926: 63 (*Neoduvalius*, TL: Prenj-planina in cca. 1400 m)

 Distribution: Bosnia & Herzegovina (Prenj Mts)

**9 *Duvalius (Neoduvalius) klimeschi*** Winkler, 1914: 171 (*Trechus*, TL: Troglavgebiet (Dinarische Alpen) an der dalmatinisch-bosnischen Grenze in zirka 1500 Meter Höhe)

 Distribution: (Croatia, Bosnia & Herzegovina)–Mt. Troglav

**10 *Duvalius (Neoduvalius) kodrici*** Scheibel, 1938: 221 (*Neoduvalius*, TL: Plitvička–Plješevica, Kočevlje (Gotschee))

 Distribution: Croatia–Mala Kapela Mts, Plješevica Mts (Pretner 1973: 171)

**11 *Duvalius (Neoduvalius) langhofferi*** Csiki, 1913: 386 (*Anophthalmus*, TL: Croatia: in antro prope Josipdol a Dom [=Špilja u Mekoti (=Plandište jama) (Bedek et al. 2006: 73)])

 Distribution: Croatia (Ogulin)

**12 *Duvalius (Neoduvalius) neumanni*** Müller, J., 1911 1 (*Trechus*, TL: Höhle „Dragišica“ bei Petrovac (Bosnien) [=Dragišići pećina])

 Distribution: Bosnia & Herzegovina (Bosanski Petrovac, Dragišići)

**13 *Duvalius (Neoduvalius) opermanni*** Scheibel, 1933: 241 (*Duvalius*, TL: Höhle nächst Rakovica in Kroatien [=Dumenčića špilja (Bedek et al. 2006: 21)])

 Distribution: Croatia (Rakovica, Slunj)

**14 *Duvalius (Neoduvalius) petraeus*** Knirsch, 1927: 51 (*Neoduvalius*, TL: Muharnica-planina. Süd.-Bosn.)

 Distribution: Bosnia & Herzegovina (Muharnica Mts)

**15 *Duvalius (Neoduvalius) reitteri*** Miller, 1881: 203 (*Anophthalmus*, TL: Grotte bei Mogorice, Südkroatien, im Likaner Reg. [=Pčelina špilja (Buljmize, Mogorić, Gospić) (Bedek et al. 2006: 46)])

 = *Duvalius (Neoduvalius) acherontius* Schaufuss, 1881: 36 (*Anophthalmus*,TL: in cavernis Croatiae) synonymy in Heyden, 1883: 10

 Distribution: Croatia (Lika), Bosnia & Herzegovina (Bosnia)

**16 *Duvalius (Neoduvalius) schatzmayri*** Müller, J., 1912: 297 (*Trechus*, TL: Höhle des Prologgebirges an der bosnisch-dalmatinischen Grenze [=Mračna pećina (Pretner 1973: 172)])

 Distribution: Bosnia & Herzegovina/Croatia (Dalmatia ): Prolog Mts

**17 *Duvalius (Neoduvalius) starivlahi***Guéorguiev, B.V., Ćurčić, S.B. & Ćurčić, B.P.M., 2000: 227 (*Duvalius*, TL: Hadži-Prodanova Pećina Cave, v. Raščići, near Ivanjica, cca 650 m)

 Distribution: Serbia (Ivanjica)

**18 *Duvalius (Neoduvalius) styx*** Apfelbeck, 1904: 139 (*Trechus*, TL: Bosnien, Höhle bei Vacar Vakuf (Zentral-Bosnien))

 Distribution: Bosnia & Herzegovina (Mrkonjic Grad), Croatia (Mala Kapela Mts, Plitvicka jezera, Medvedjak) (Pretner 1973: 172)

**19 *Duvalius (Neoduvalius) vranensis*** Breit, 1904: 28 (*Trechus*, TL: Höhle des Vran-Gebirges in der Herzegovina [=Mijatova jama (Hauser, 2011: 117)])

 Distribution: Bosnia & Herzegovina (Vran Mts)

**20 *Duvalius (Neoduvalius) zlatiborensis*** Ćurčić S. B., Brajković, Ćurčić, B. P. M., 2005: 65 (*Rascioduvalius*, TL: Markova (=Ršumska) Pećina cave, village of Gornji Ljubiš, Mt. Zlatibor, Western Serbia)

 Distribution: Serbia (Užice, Zlatibor Mts)

 Note: This species was described based on two females collected in Markova pećina cave, which is very close (ca 10 km) to Stopića pećina cave, type locality for *Duvalius (Neoduvalius) cvijici stopicensis* Jeannel and ca 2 km to Murtenica planina Mts., type locality for *Duvalius (Neoduvalius) cvijici cvijici* Jeannel. Based on presented morphological differencies it is very probable that all these taxa are conspecific, inhabiting both caves and MSS of the region of Zlatibor and Murtenica Mts.

## Supplementary Material

XML Treatment for Duvalius (Neoduvalius) gejzadunayi
